# Copy Number Variation and Structural Genomic Findings in 116 Cases of Sudden Unexplained Death between 1 and 28 Months of Age

**DOI:** 10.1002/ggn2.202200012

**Published:** 2022-11-07

**Authors:** Catherine A. Brownstein, Elise Douard, Robin L. Haynes, Hyun Yong Koh, Alireza Haghighi, Christine Keywan, Bree Martin, Sanda Alexandrescu, Elisabeth A. Haas, Sara O. Vargas, Monica H. Wojcik, Sébastien Jacquemont, Annapurna H. Poduri, Richard D. Goldstein, Ingrid A. Holm

**Affiliations:** ^1^ Robert's Program on Sudden Unexplained Death in Pediatrics Boston Children's Hospital Boston MA 02115 USA; ^2^ Division of Genetics and Genomics Boston Children's Hospital Boston MA 02115 USA; ^3^ The Manton Center for Orphan Disease Research Boston Children's Hospital Boston MA 02115 USA; ^4^ Department of Pediatrics Harvard Medical School Boston MA 02115 USA; ^5^ Sainte Justine Hospital Research Center Montreal Quebec H3T 1C5 Canada; ^6^ Department of Neuroscience University of Montreal Montreal Quebec H3C 3J7 Canada; ^7^ Department of Pathology Boston Children's Hospital and Harvard Medical School Boston MA 02115 USA; ^8^ Epilepsy Genetics Program Department of Neurology Boston Children's Hospital Boston MA 02115 USA; ^9^ F. M. Kirby Neurobiology Center Department of Neurology Boston Children's Hospital Boston MA 02115 USA; ^10^ Department of Neurology Harvard Medical School Boston MA 02115 USA; ^11^ Department of Genetics Harvard Medical School Boston MA 02115 USA; ^12^ Department of Medicine Brigham and Women's Hospital Harvard Medical School Boston MA 02115 USA; ^13^ The Broad Institute 415 Main St Cambridge MA 02142 USA; ^14^ Department of Research Rady Children's Hospital‐San Diego San Diego CA 92123 USA; ^15^ Division of Newborn Medicine Department of Pediatrics Boston Children's Hospital Boston MA 02115 USA; ^16^ Division of General Pediatrics Department of Pediatrics Boston Children's Hospital Boston MA 02115 USA; ^17^ Department of Pediatrics University of Montreal Quebec H3T 1C5 Canada

**Keywords:** autism spectrum disorder, chromosomal microarray, death, genetics, genomics, sudden infant death syndrome, sudden unexplained death in childhood

## Abstract

In sudden unexplained death in pediatrics (SUDP) the cause of death is unknown despite an autopsy and investigation. The role of copy number variations (CNVs) in SUDP has not been well‐studied. Chromosomal microarray (CMA) data are generated for 116 SUDP cases with age at death between 1 and 28 months. CNVs are classified using the American College of Medical Genetics and Genomics guidelines and CNVs in our cohort are compared to an autism spectrum disorder (ASD) cohort, and to a control cohort. Pathogenic CNVs are identified in 5 of 116 cases (4.3%). Variants of uncertain significance (VUS) favoring pathogenic CNVs are identified in 9 cases (7.8%). Several CNVs are associated with neurodevelopmental phenotypes including seizures, ASD, developmental delay, and schizophrenia. The structural variant 47,XXY is identified in two cases (2/69 boys, 2.9%) not previously diagnosed with Klinefelter syndrome. Pathogenicity scores for deletions are significantly elevated in the SUDP cohort versus controls (*p* = 0.007) and are not significantly different from the ASD cohort. The finding of pathogenic or VUS favoring pathogenic CNVs, or structural variants, in 12.1% of cases, combined with the observation of higher pathogenicity scores for deletions in SUDP versus controls, suggests that CMA should be included in the genetic evaluation of SUDP.

## Graphical abstract

Chromosomal microarray (CMA) data for 116 sudden unexplained death in pediatrics (SUDP) cases is compared to an autism spectrum disorder (ASD) cohort and a control cohort. Pathogenicity scores for deletions are significantly elevated in the SUDP cohort versus controls (*p* = 0.007) and are not significantly different from the ASD cohort. This data suggests that CMA should be included when evaluating SUDP.

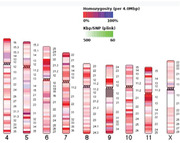

## 1 Introduction

Sudden unexpected death in pediatrics (SUDP) includes sudden infant death syndrome (SIDS) and sudden unexplained death in childhood (SUDC).^[^ ^1^ ^]^ The broader term of SUDP encompasses unexpected deaths in apparently healthy children less than 1 year of age (SIDS), or older than 1 year of age (SUDC), that remains unexplained despite a complete autopsy, death scene investigation, and ancillary testing, though most sudden, unexpected pediatric mortality occurs before the age of 6 months.^[^ ^2^ ^]^ The etiological model of SUDP includes extrinsic (e.g., sleep position, bed‐sharing, tobacco exposure) and intrinsic (e.g., male sex, race, prematurity) factors that together contribute to SUDP. Evidence for a genetic basis in SUDP includes studies using exome sequencing (ES) that have identified a potential genetic cause in up to 20% of SIDS cases,^[^ ^3^, ^4^ ^]^ including our finding of clinically informative variants in 11% of SIDS and SUDC cases.^[^ ^5^ ^]^ Variants have been identified in genes related to epilepsy,^[^ ^6^, ^7^, ^8^ ^]^ cardiac channelopathies,^[^ ^4^ ^]^ and metabolic diseases^[^ ^9^ ^]^ (see refs. [10, 11] for reviews).

Another important genetic mechanism leading to disease is structural variation, including copy number variants (CNVs), which are deletions or duplications usually 1 kb in size or larger. Although CNVs are present in most individuals in the population and frequently not associated with disease, CNVs have been implicated in a number of conditions, including autism spectrum disorder (ASD),^[^ ^12^, ^13^, ^14^ ^]^ developmental disabilities,^[^ ^15^, ^16^ ^]^ attention deficit hyperactivity disorder,^[^ ^17^ ^]^ schizophrenia,^[^ ^18^ ^]^ Crohn's disease,^[^ ^19^ ^]^ epilepsy,^[^ ^20^, ^21^ ^]^ and multiple congenital anomalies.^[^ ^22^, ^23^, ^24^ ^]^ In the clinical setting, chromosomal microarray (CMA) analysis is included in the initial evaluation of individuals with ASD,^[^ ^25^, ^26^ ^]^ developmental disabilities,^[^ ^27^, ^28^ ^]^ multiple congenital anomalies,^[^ ^22^ ^]^ and epilepsy,^[^ ^27^, ^29^, ^30^ ^]^ to identify CNVs with established disease associations. Additionally, CNVs with potential clinical significance have been detected by CMA analysis in 12–15% of stillbirth cases.^[^ ^31^, ^32^, ^33^, ^34^, ^35^, ^36^ ^]^ However, little is known about the role CNVs, and the genes contained within them, may play in SUDP.^[^ ^37^ ^]^ One study reported CNVs in 3/27 SIDS cases (11%), including two de novo CNVs at chromosome 6p22, one a 1.9 Mb deletion and the other a 240 kb duplication; a third case had an 8q24.3‐qter duplication and a 22q13.3‐qter deletion due to an unbalanced translocation.^[^ ^38^ ^]^

It is hypothesized that unexplained stillbirth, SIDS, and SUDC represent a continuum of as yet unexplained death from fetal life through childhood, with some shared genetic associations already reported involving single nucleotide variants in single genes.^[^ ^28^, ^39^ ^]^ A recently reported association of CNVs with stillbirth suggests that CNVs may play a role in SIDS and SUDC^[^ ^40^ ^]^ and another concluded that SUDC genetic architecture may have some overlap with that of neurodevelopmental disorders.^[^ ^8^ ^]^ To assess the role of CNVs in SIDS and SUDC, we performed CMA analyses in a cohort of SUDP cases and report on the yield and the types of CNVs identified. In addition, we performed a comparison analysis of our cohort versus a control cohort. We also compared our cohort to an independent cohort with ASD, a condition with well‐established CNV associations.^[^ ^25^, ^26^ ^]^ Such analysis allows us to investigate potential similarity in the genetic architectures of both disorders, and to provide further recommendations about the diagnosis, therapeutic choices, and pediatric follow‐up of infants with a CNV.

## 2 Experimental Section

### 2.1 Cases

Cases were infants and children who died from SUDP, and cases were either obtained from the San Diego Office of the Medical Examiner or referred to Robert's Program on SUDP at Boston Children's Hospital (BCH) by parents, physicians, medical examiners, and researchers. Informed consent was obtained from parents of cases in Robert's Program; cases from the San Diego Office of the Medical Examiner were obtained in accordance with the California Code, Section 27491.41 for research in SIDS. The study was approved by the BCH Institutional Review Board (IRB‐P00011014)). A detailed phenotypic analysis of each case was performed from available data, including the circumstances of death (e.g., risk factors such as bed‐sharing); coincident acute illness; general medical history, neurological history; growth and development; physical findings; obstetric and birth histories; and family history including sudden death, febrile seizures, epilepsy, neuro‐developmental disorders, and cardiac disorders. When available, detailed neuropathological analysis includes review of materials for specific abnormalities found to be associated with SUDP, including abnormalities of the hippocampus.^[^ ^41^, ^42^ ^]^ ES had previously been performed on all cases, and the results were available for this study.^[^ ^5^ ^]^

### 2.2 CMA Analysis

Genomic DNA was extracted from tissue specimens or blood using standard protocols (Qiagen, Valencia, CA) and MagNA Pure Compact System (Roche Diagnostics, Pleasanton, CA). Dye‐swap array‐CGH (comparative genomic hybridization) experiments were performed according to experimental procedures described by Agilent Technologies (Santa Clara, CA) using Agilent 4 × 180 K Surescan arrays. Data analysis was performed using Agilent Cytogenomics Software (Agilent Cytogenomics v3.0.6.6). CNV coordinates were based on the National Center for Biotechnology Information (NCBI) Genome Reference Consortium Human Build 37, GRCh37 (hg19). The absence of heterozygosity (AOH) regions was detected using the Agilent loss of heterozygosity (LOH) algorithm.

CNVs were classified as pathogenic or likely pathogenic (P/LP) or variant of uncertain significance (VUS) according to the American College of Medical Genetics and Genomics guidelines.^[^ ^26^, ^43^ ^]^ Using the publicly available tool Franklin by Genoox (https://www.genoox.com/the‐genoox‐platform/), CNVs that were VUS favoring pathogenic were additionally identified, meaning that despite uncertainties, some scientific evidence supportive of pathogenicity existed.

Variants with reference to case‐specific phenotypic data were reviewed in ClinVar (https://www.ncbi.nlm.nih.gov/clinvar/). The chromosomal intervals were also confirmed in the UK Biobank, Human Gene Mutation Database, Databases of Genomic Variants (DGV), and the Genome Aggregation Database (gnomAD)^[^ ^44^, ^45^, ^46^ ^]^ to confirm rarity and avoid misclassifications. Genes within CNV intervals were assessed for pLi (probability of loss‐of‐function intolerance).^[^ ^47^ ^]^ All of the cases had previously undergone ES^[^ ^5^ ^]^ and the ES data were analyzed for the possibility of a loss of function (LOF) variant on the other allele by querying the ES data for rare variants in genes within the CNV interval using Codified Genomics and WuXi Nextcode browsers. Loss of heterozygosity (LOH) intervals was reported if over 10 Mb in size.

### 2.3 Controls and ASD Populations

**Figure 1:**
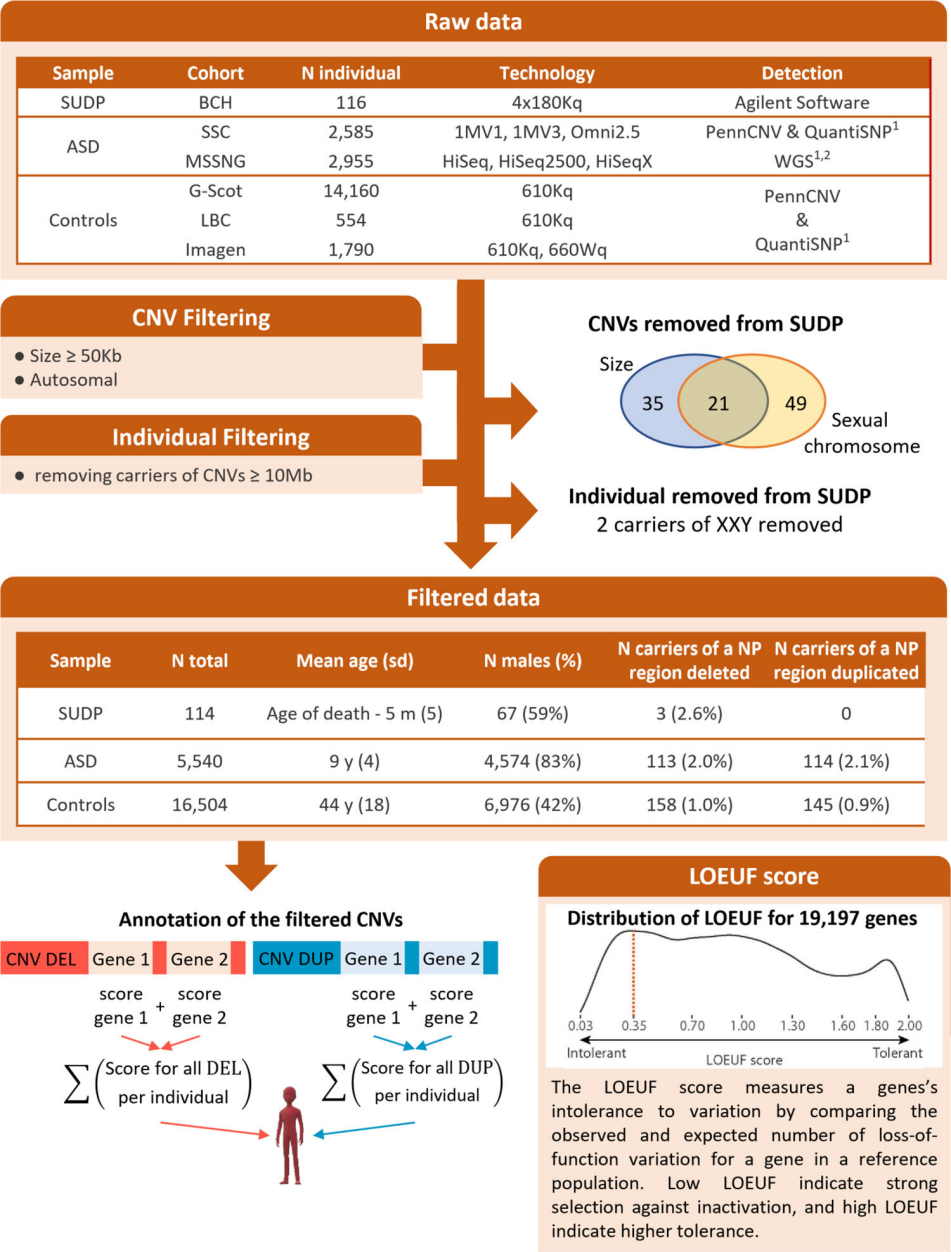
Methodological pipeline for the CNV filtering and annotation. The two tables describe the samples of SUDP, ASD, and UP populations before and after filtering. The Venn diagram represents the distribution of the SUDP CNVs discarded in the function of the filtering criteria to which they belong: The size is less than 50 Kb and it is positioned on a sex chromosome. The last density plot represents the distribution of LOEUF score across 19 197 coding genes. A LOEUF score ≤0.35 is the defined clinical threshold for intolerant genes. For CNV annotation, the coding gene totally encompassed in deletions and duplications were identified and the LOEUF score of each gene was attributed. For each individual, the number of genes encompassed and the 1/LOEUF scores for deletions and duplications (referred to as the “pathogenicity score”) were summed separately. CNV: copy number variant; N: number; NP: neuropsychiatric; DEL: deletions; DUP: duplications; SUDP: Sudden Unexplained Death in Pediatrics; BCH: Boston children hospital; ASD: autism spectrum disorders; SSC: Simons Simplex Collection; UP: unselected populations; G‐Scot: Generation Scotland; LBC: Lothian birth cohort; LOEUF: loss‐of‐function observed/expected upper bound fraction. ^1^Huguet et al., *Mol. Psy*. (2021); ^2^Trost et al., *Am. J. Hum. Genet*. (2018).

Three pooled unselected community‐based cohorts were included as controls: IMAGEN (*N* = 1,802),^[^ ^48^ ^]^ Generation Scotland (GS)^[^ ^49^ ^]^ (*N* = 14,160), and the Lothian Birth Cohort^[^ ^50^ ^]^ (LBC) (*N* = 554) (see **Figure** **1**). Children with ASD from two pooled cohorts were also included: the Simons simplex collection (SSC)^[^ ^51^ ^]^ (*N* = 2,585) and the MSSNG database.^[^ ^52^ ^]^

A previously published pipeline was used for the detection and filtering of the CNVs from these additional cohorts.^[^ ^53^ ^]^ To harmonize the SUDP, control, and ASD samples, all CNVs <50 Kb were discarded and individuals with a CNV ≥10 Mb were removed (see Figure 1). Studies for each cohort were reviewed by local IRBs.^[^ ^48^, ^49^, ^50^, ^51^, ^52^ ^]^

#### 2.3.1 Genome‐Wide CNV Pathogenicity Analyses

For each individual, a pathogenicity score for deletions and duplications was computed separately using a previously published annotation pipeline (see Figure 1).^[^ ^53^ ^]^ For the pathogenicity score, the loss‐of‐function observed/expected upper bound fraction (LOEUF) score was used, which was a measure of haploinsufficiency available for 19,197 genes. It was defined as the estimated upper limits of the confidence interval for the observed to expected loss‐of‐function variants ratio. Each coding gene with all isoforms fully encompassed in filtered CNVs was identified using ENSEMBL map (Gencode V19 (hg19))^[^ ^54^ ^]^ and was annotated using the inverted LOEUF (1/LOEUF) score (gnomAD,^[^ ^46^ ^]^ which ranges from 0.5 (gene tolerant to haploinsufficiency) to 33.3 (gene intolerant to haploinsufficiency). A score of 0 was assigned to individuals with no coding genes encompassed in any CNV. The genome‐wide CNV burden was tested with a logistic regression model as follows:

(1) $$\begin{array}{ccc} \ln(\mathrm{odds}(Y_{i} & = & {\mathrm{diagnosis}}_{i}))\approx \beta_{0}+\beta_{1}\;{\mathrm{Pathogenicity}\;\mathrm{Score}}_{\mathrm{DEL}i}\\ & & +\;\beta_{2}\;{\mathrm{Pathogenicity}\;\mathrm{Score}}_{\mathrm{DUP}i}+\beta_{3}{\mathrm{sex}}_{i} \end{array}$$

where PathogenicityScore_DEL/DUP_ are the sum of 1/LOEUF for deletions (DEL) and duplications (DUP) respectively. *β*_0_, *β*_1_, *β*_2_, and *β*_3_ are the vectors of coefficients for fixed effects.

#### 2.3.2 Neuropsychiatric Loci Analyses

Recurrent CNVs are defined as genomic loci flanked by low copy repeat sequences that greatly increase the risk of homologous recombination. These non‐allelic homologous recombinations result in similar or identical CNVs in unrelated individuals.^[^ ^53^ ^]^

Prior studies have identified 47 recurrent CNVs and genes associated with neurodevelopmental or neuropsychiatric disorders (see Table S1, Supporting Information).^[^ ^55^ ^]^ These regions were annotated in the SUDP, control, and ASD samples if an identified deletion or duplication overlapped at ≥40% with a neuropsychiatric CNVs or if a gene associated with neuropsychiatric disorders was disrupted.^[^ ^53^ ^]^ To test for differences in the prevalence of variants in neuropsychiatric loci between cohorts, two‐sided Fisher's exact tests were employed. *P*‐values were adjusted for multiple comparisons using Benjamini‐Hochberg correction for false discovery rate (FDR).

To address the possibility that bed‐sharing resulted in accidental suffocation of a subset of the cohort, the above analyses were repeated removing the bed‐sharing cases.

## 3 Results

### 3.1 Study Cohort

We conducted CMA analysis for 116 SUDP cases. The demographics of the cohort are summarized in **Table** **1**. Average age at death was 5.7 months (SD 5.8 months) and ranged from 1.5 to 28 months; 69/116 (59%) of cases were boys. Average gestational age was 38.3 weeks. 34/116 (30%) of cases were co‐sleeping at the time of death, and 38/116 (43%) of cases were found in the prone position. Three cases had a history of febrile seizures. **Tables** **2** and **3** summarize the demographics and circumstances around death, respectively, for the cases in which P/LP or VUS favoring pathogenic results were found.

**Table 1:** Demographics of 116 cases of SUDP cohort

Total SUDP cohort with CMA data (*n* = 116)	Number of probands with available information	Proportion
**Age at death**	116	
<2 months	17	15%
2–<6 months	67	58%
6–<12 months	22	19%
≥12 months	10	9%
**Sex**	116	
Male	69	59%
Female	47	41%
**Ancestry via PCA**	116	
European	61	53%
African American	9	8%
East Asian	10	9%
Mixed race	36	31%
**Gestational age**	108	
≥37 weeks	87	81%
34–37 weeks	12	10%
<34 weeks	9	8%
**Position found (for children <1 year old, who died during sleep)**	88	
Prone	38	43%
Supine	33	38%
Side	13	15%
Upright/partially upright	4	5%
**Sleep site**	99	
Crib	41	41%
Adult bed	37	37%
Couch	8	8%
Car seat	2	2%
Held	3	3%
Other	8	8%
**Sleeping circumstances**	112	
Shared sleep surface	34	30%
Sleeping alone	78	70%
**Personal history**		
Antecedent fever	8	
Antecedent minor illness	29	
Febrile seizures	3	
Other seizures	1	
Low birth weight (<2500 g)	17	
ROSC	8	
**Family history**		
SIDS or SUDC	10	
SCD <50 years old	3	
Febrile seizures	11	
Seizures	8	
Syncope (excluding vasovagal)	3	

**Table 2:** Demographics of the 18 SUDP cases with pathogenic (5 cases) or VUS favoring pathogenic (13 cases) CMA results

Case	Sex	Ancestry	Age at death (in months)	Low birth weight [g]
1	F	European	3	N:2948
2	F	European	5	U
3	F	European	3	N
4	M	Mixed	3.5	N
5	M	Mixed	1.4	Y:2300
6	M	Mixed	11	U
7	M	Mixed	1.25	U
8	M	East Asian	11	N/A
9	M	Mixed	4	N
10	F	European	2	N
11	F	European	6	Y:1814
12	F	European	23	U
13	F	African American	3.5	N:3255
14	M	Asian	5	N
15	M	Mixed	1.5	Y:2300
16	M	European	3	Y:2293
17	F	European	2	N/A

**Table 3:** Circumstances around death for the 18 SUDP cases with pathogenic (5 cases) and VUS Favoring pathogenic (13 cases) CMA results

Case	Position found	Sleep site	Sleep circumstances	Fever	Minor illness
1	Prone	Pack n play	Alone	N	Y
2	Supine	Adult bed	Alone	U	U
3	Prone	Crib	Alone	N	N
4	Prone	Crib	Alone	N	N
5	Supine	Adult bed	Cosleeping	N	N
6	U	U	U	U	U
7	Supine	Adult bed	Alone	U	U
8	Supine	Sofa	Cosleeping	Y	Y
9	Supine	Couch	Cosleeping	N	N
10	Supine	Crib	Alone	N	Y
11	Supine	Adult bed	U	N	N
12	U	Crib	Alone	N	N
13	Supine	Other	Cosleeping	N	N
14	N/A awake	Awake in crib	Alone	N	Y
15	Supine	Adult bed	Cosleeping	N	Y
16	Prone	Adult bed	Alone	N	N
17	Prone	Adult bed	Alone	N	N

U = unknown

### 3.2 CNVs Detected

#### 3.2.1 Pathogenic CNVs

Five of the 116 SUDP cases (4.3%) had CNVs categorized as P/LP and associated with a known disease, though none of these diseases have previously been associated with SUDP. In addition, none of the associated diseases were known to be present in the children harboring the CNVs prior to their death (**Table** **4**).

**Table 4:** CNVs scored as pathogenic by Genoox

Case no.	CNV location	CNV coordinates	Size [Mb]	Type	Gene involved	Disease associated	Notes	Evidence	Carrier frequency (approximate, gnomAD)
1	chr2p16.3	chr2: 50802121–50909824	0.107 (107.7 kb)	Het deletion	NRXN1	Autism and seizures, Pitt‐Hopkins‐like syndrome 2, chromosome 2p16.3 deletion syndrome	11 Mb LOH on chromosome 8 (chr8p11.21‐q11.21, encompassing *ZMAT5*, *SFRP1*, and *MIR54A0*)	Overlap with established Hi genes or genomic regions 2E (+0.90) Both breakpoints are within the coding region of same gene (gene‐level sequence variant)	4.60e‐5
2	chr4q31.22	chr4: 147605042–147953051	348 kb	Hom deletion	TTC29	Spermatogenic failure and Non‐syndromic male infertility due to sperm motility disorder		Overlap with established Hi genes or genomic regions 2A (+1.00) Complete overlap of an established HI gene/genomic region	4.60e‐5
3	chr5q13.2	chr5:68849594–70636824	1.79 Mb	Het deletion	Several, see Table S2, Supporting Information	Spinal muscular atrophy, pseudo‐TORCH syndrome	Carrier; heterozygous deletion. Phenotype producing in compound heterozygous or homozygous state.	Overlap with established Hi genes or genomic regions 2A (+1.00) Complete overlap of an established HI gene/genomic region	2.19e‐1
4	chrXp22.33	chrX: 219609–1019952	800 kb	Het deletion	PLCXD1, GTPBP6, LINC00685, PPP2R3B, and SHOX	Short stature, wide phenotypic spectrum, Leri–Weill dyschondrosteosis, Langer mesomelic dysplasia, SHOX‐related short stature		Overlap with established Hi genes or genomic regions 2A (+1.00) Complete overlap of an established HI gene/genomic region	9.70e‐5
5	chr15q15.3	chr15:43851548–43949572	98 kb	Het deletion	PPIP5K1, CKMT1B, STRC, CATSPER2, PPIP5K1P1‐CATSPER2	Deafness‐infertility syndrome and autosomal recessive nonsyndromic deafness	Carrier; heterozygous deletion. Phenotype producing in compound heterozygous or homozygous state.	Overlap with established Hi genes or genomic regions 2A (+1.00) Complete overlap of an established HI gene/genomic region	1.07e‐2

Case 1 was a previously healthy 3‐month‐old girl who died during sleep and was found in the prone position; she had no history of seizures. Neuropathological analysis revealed findings in the dentate gyrus reported to be present in SUDP as well as temporal lobe epilepsy.^[^ ^56^ ^]^ She had a heterozygous deletion at 2p16.3 (chr2: 50802121–50909824, 107.7 kb) encompassing the gene *NRXN1*. Deletions at 2p16.3 have variable expressivity and incomplete penetrance and have been associated with susceptibility to developmental delay, ASD, schizophrenia, and dysmorphic features.^[^ ^57^ ^]^ We previously reported a case with epilepsy and impaired respiratory drive leading to death and single nucleotide variants in both *NRXN1* and *NRXN2* categorized as VUS.^[^ ^58^ ^]^ None of the characteristic facial dysmorphisms of NRXN1 deletion were noted in case 1, though they may not be well‐developed in a 3‐month‐old.

In addition to the 2p16.3 deletion, case 1 also had an 11 Mb region of LOH at 8p11.21‐q11.21 (chr8:40438757–51547523) encompassing *ZMAT5*, and *SFRP1*, neither of which are known to be associated with any disease. Previous ES data showed no rare variants in the genes in this LOH region in this case.

Case 2 was a previously healthy 5‐month‐old girl found in the supine position. She had a homozygous deletion at 4q31.22 (chr4:147605042–147953051, 348 kb), which included *TTC29*. Homozygous and compound heterozygous P/LP variants in *TTC29* are associated with spermatogenic failure and non‐syndromic male infertility due to a sperm motility disorder, a phenotype unrelated to SUDP and not relevant in females.^[^ ^59^, ^60^ ^]^

Case 3 was a previously healthy 3‐month‐old girl found in the prone position. She had a heterozygous deletion at chr5q13.2 (chr5:68849594–70636824, 1.79 Mb) encompassing several genes including *SMN2* and *OCLN* (see Table S1, Supporting Information). Homozygous or compound heterozygous deletions of this interval are associated with spinal muscular atrophy (SMA) and P/LP variants are associated with pseudo‐TORCH syndrome.^[^ ^61^ ^]^ There is no known phenotype associated with heterozygous deletions of this region.

Case 4 was a previously healthy 3.5‐month‐old boy found in the prone position. He had a deletion at chrXp22.33 (chrX:219609–1019952, 800 kb) encompassing *PLCXD1*, *GTPBP6*, *LINC00685*, *PPP2R3B*, and *SHOX*. The only disease gene is *SHOX*, and SHOX haploinsufficiency leads to a wide phenotypic spectrum that includes syndromic and non‐syndromic short stature, a phenotype unrelated to SUDP.^[^ ^62^ ^]^ Of note, at autopsy, the infant was noted to be below the second percentile for length and below the fifth percentile for weight.

Case 5 was a previously healthy 6‐week‐old boy who was found in the supine position while co‐sleeping in an adult bed. He had a heterozygous deletion at chr15q15.3 (chr15:43851548–43949572, 98 kb) encompassing *PPIP5K1*, *CKMT1B*, *STRC*, *CATSPER2*, and *PPIP5K1P1‐CATSPER2*. Homozygous deletion of *STRC* and *CATSPER2* are associated with autosomal recessive deafness‐infertility syndrome DFNB16.^[^ ^63^ ^]^ Review of the ES data did not reveal P/LP variants in any of these genes in this case.^[^ ^5^ ^]^ There is no known phenotype associated with heterozygous carriers.

#### 3.2.2 CNVs Categorized as VUS Favoring Pathogenic

Nine cases (7.8%) had one or more CNVs categorized as a VUS favoring pathogenic (see **Table** **5**).

**Table 5:** CNVs scored as VUS favoring pathogenic by Genoox

Case No.	CNV Location	CNV Coordinates	Size (Mb)	Type	Gene involved	Disease associated	Notes/ Presumed causative finding on ES	Evidence	Carrier frequency (approximate, gnomAD)
6	chr15q11.2	chr15:22835886–23080961	0.245 (245 kb)	Het deletion	TUBGCP5, CYFIP1, NIPA2, NIPA1	15q11.2 deletion syndrome		Overlap with established Hi genes or genomic regions 2A (+0.75)	1.89e‐3
7	chr15q11.2	chr15:22835886–23082821	0.247 (247 kb)	Het deletion	TUBGCP5, CYFIP1, NIPA2, NIPA1	15q11.2 deletion syndrome		Overlap with established Hi genes or genomic regions 2A (+0.75)	1.80e‐3
8	chr13q34	chr13:111181035–111321385	0.140 (140 kb)	Het duplication	RAB20, NAXD, and CARS2	Developmental delay	Also has pathogenic PDHA1 c.1246C>T, p.Arg416Cys	Breakpoint(s) within established HI genes 2K (+0.30)	4.60e‐5
19	chr2p21	chr2: 45168836–45171619	0.028 (2.78 kb)	Het duplication	SIX3‐AS1, SIX3	No disease association		Breakpoint(s) within established HI genes 2K (+0.30)	4.60e‐5
	chr22q11.21	chr22: 19744894–19758306	0.134 (13.4 kb)	Het duplication	TBX1	No disease association		Breakpoint(s) within established HI genes 2K (+0.30)	0
10	chr2p23.1	chr2: 30102783–30276858	0.174 (174 kb)	Het duplication	ALK	No disease association		Breakpoint(s) within established HI genes 2K (+0.30)	1.38e‐4
11	chr7q36	chr7: 158424788–158651068	0.226 (226 kb)	Het duplication	NCAPG2, ESYT2, WDR60	No disease association		Breakpoint(s) within established HI genes 2K (+0.30)	4.60e‐5
12	chr8p23.2	chr8: 3379054–3602403	0.223 (223 kb)	Het deletion	CSMD1	Epilepsy and seizures		Both breakpoints are within the coding region of same gene (gene‐level sequence variant) 2E (+0.45)	1.38e‐4
13	chr13q32.3	chr13: 100635361–100752793	0.117 (117 kb)	Het duplication	ZIC2, LINC00554, LOC105370333, PCCA	No disease association		Breakpoint(s) within established HI genes 2K (+0.30)	9.50e‐5
14	chr22q13.33	chr22: 51063762–51066512	0.275 (2.75 kb)	Het duplication	ARSA	Metachromatic leukodystrophy due to Arylsulfatase A enzyme deficiency		Breakpoint(s) within established HI genes 2K (+0.30)	1.84e‐4

Cases 6 (an 11‐month‐old boy) and 7 (a 5‐month‐old boy) both had a 15q11.2 deletion (chr15:22835886–23080961, 245 kb, and chr15:22835886–23082821, 246 kb, respectively) encompassing *TUBGCP5, CYFIP1, NIPA2*, and *NIPA1* and associated with 15q11.2 deletion syndrome (Burnside‐Butler syndrome), a neurodevelopmental disorder characterized by changes in brain morphology, behavior, and cognition).^[^ ^64^ ^]^ Deletions and duplications of this region are associated with a variety of neurodevelopmental features, including developmental delay, ASD, behavioral problems, and seizures.^[^ ^65^, ^66^ ^]^ The 15q11.2 region is also a schizophrenia susceptibility region.^[^ ^67^, ^68^ ^]^ Case 7 had evidence for bilamination of the hippocampus on autopsy.^[^ ^42^ ^]^ Both cases 6 and 7 were specifically noted to be not dysmorphic on autopsy. The developmental histories of the two cases are unknown.

Case 8, an 11‐month‐old boy found in the supine position while co‐sleeping on a sofa, had a duplication at chr13q34 (chr13:111181035–111321385, 140 kb) encompassing *RAB20, NAXD*, and *CARS2*. A similar duplication in this interval was reported in ClinVar in a patient with a phenotype categorized as “developmental delay and/or other significant developmental or morphological phenotypes” was scored as a VUS;^[^ ^69^ ^]^ there are no additional publications that confirm this association. On ES we previously found a known pathogenic *PDHA1* (chrXp22.12) c.1246C>T, p.Arg416Cys variant responsible for X‐linked Pyruvate Dehydrogenase E1‐alpha deficiency that had been previously published with functional data.^[^ ^5^, ^70^ ^]^ The family history of case 8 is unknown.

Case 9, a 4‐month‐old boy found supine on a couch co‐sleeping, had two VUS favoring pathogenic CNVs. The first was a chr2p21 duplication (chr2:45168836–45171619, 2.8 kb) encompassing *SIX3* and *SIX3‐AS1*. Heterozygous pathogenic variants in *SIX3* are associate with holoprosencephaly.^[^ ^71^ ^]^ The second CNV was a duplication at chr22q11.21 (chr22:19744894–19758306, 13.4 kb) and encompassing *TBX1*, which is considered to be responsible for features velocardiofacial syndrome in the presence of haploinsufficiency.^[^ ^72^ ^]^ Duplication of chr2p21 or chr22q11.21 is not known to be associated with a phenotype.

Case 10 was a 2‐month‐old girl found supine in a crib and had a chr2p23.1 duplication (chr2:30102783–30276858, 174 kb) encompassing ALK. ALK is a receptor tyrosine kinase linked to neuroblastoma and lung cancer susceptibility.^[^ ^73^ ^]^

Case 11, a 6‐month‐old girl found supine on an adult bed and noted on past medical history to have a low birth weight but found to be at the 50–75th percentile at autopsy, had a chr7q36.3 duplication (chr7: 158424788–158651068, 226 kb) encompassing *NCAPG2*, *ESYT2*, and *WDR60*. Duplications at chr7q36.3are not known to be associated with any condition.

Case 12, a 23‐month‐old girl, had a personal and family history of febrile seizures. End‐folium sclerosis of the hippocampus, a morphological abnormality reported in association with temporal lobe epilepsy, was noted on autopsy. She had a chr8p23.2 deletion (chr8: 3379054–3602403, 223 kb) encompassing *CSMD1*. She also had a splice region variant previously detected by ES on the other *CSMD1* allele (chr8:3611570G>C, NM_033225.5:c.819‐6C>G), which is common and found in 6.3% of Ashkenazi in gnomAD. There is a case report of a t(4;8)(p15.2;p23.2) translocation interrupting the coding sequence of *CSMD1* segregating with juvenile myoclonic epilepsy, self‐limited photosensitive occipital epilepsy, and migraine with aura in a family.^[^ ^74^ ^]^ An 8p23‐p21 deletion encompassing *CSMD1* was found in a Saudi Arabian family with autosomal dominant epilepsy and intellectual disability.^[^ ^75^ ^]^

#### 3.2.3 CNVs Detected over 1 Mb in Size and LOH

In total, a CNV was detected in 90 cases (78% of total cohort). Twenty‐one cases (18% of total cohort, 23% of those with a CNV) had a CNV greater than 1 Mb in size (Table S2, Supporting Information). Eleven cases had a CNV deletion 1 Mb or greater (10.3%), and 12 cases had a CNV duplication 1 Mb or greater. One case had two deletions over 1 Mb in size (case 00–0608), and two cases had both a deletion and a duplication larger than 1 Mb (cases 00–0774 and 00–0288).

Three cases, 15, 16, and 17 (5‐month‐old boy, 1.5‐month‐old boy, and 3‐month‐old girl) had abnormal CMA results involving the X chromosome (see **Table** **6**). The two boy cases (15 and 16) had complete duplication of the X chromosome consistent with a 47,XXY karyotype and the diagnosis of Klinefelter syndrome. Neither case carried an antemortem diagnosis of Klinefelter syndrome, and neither had a history of seizures. Case 15 also had an 81 kb duplication at 15q26.2 encompassing *NR2F2* scored as a VUS (see below). CNV findings consistent with a 47,XXY karyotype were found in 2/69 boys in the cohort, which is greater than the general population prevalence of Klinefelter syndrome of 1/500 males (*p* = 0.04).^[^ ^76^ ^]^

**Table 6:** Structural variation and LOH on the X and Y chromosomes

Case no.	CNV location	CNV coordinates (hg19)	Size [Mb]	Interpretation	Frequency in population	Notes/presumed causative finding on ES
15	chrXp22.33	chrX:60701–2646815	2.6 Mb	chrX duplication, suggesting XXY	2.00e‐3^[^ ^76^ ^]^	Also has a chr15q26.2 deletion scored as VUS
	chrXp22.–3 ‐ p11.1	chrX:2676115–58543823	55.8 Mb			
	chrXq11–1 ‐ q28	chrX:61781601–155197079	93 Mb			
16	chrXp22.–3 ‐ p11.1	chrX: 2700316–58484662	56 Mb	chrX duplication, suggesting XXY	2.00e‐3^[^ ^76^ ^]^	
	chrXq11–1 ‐ q28	chrX:61931689–155197079	93 Mb			
17	chrXp11.3‐q12, LOH	chrX:44216570–66956221	22.7 Mb	Large LOH, suggesting UPD	2.11e‐5^[^ ^77^ ^]^	Also has LP variant in *SCN1A* c.3886T>A, chr2‐166866312‐A‐T, p.S1296T and chr11p15.5 duplication scored VUS encompassing HOTS, and H19.

The girl case (17) had a 22.7 Mb area of LOH on chromosome Xp11.3‐q12 (chrX: 44216570–66956221) suggesting partial uniparental disomy (UPD). A population‐based study of X and Y chromosome variations estimated that UPD of the X chromosome has a population prevalence of 1 in 47,305 women.^[^ ^77^ ^]^ On ES we previously found this case to have an LP variant in *SCN1A* (c.3886T>A, chr2‐166866312‐A‐T, p.S1296T)^[^ ^7^ ^]^ that may explain SUDP.^[^ ^5^ ^]^ Pathogenic *SCN1A* variants have been associated with SIDS^[^ ^7^ ^]^ and sudden unexpected death in epilepsy (SUDEP).^[^ ^78^, ^79^, ^80^ ^]^

#### 3.2.4 VUS CNVs

One or more CNVs categorized as VUS were detected in 53 cases (46%) (see **Table** **7** for selected VUS [those with a diagnostic exome finding, a CNV seen in more than one case, or a VUS over 1 Mb]). Five of these cases also had an ES finding that potentially explained their death. None of the genes involved were related to a disease that could be associated with SUDP.

**Table 7:** Selected CNVs scored as VUS

Case	Location	Coordinates (hg19)	Size	Zygosity	Genes involved	Gene‐disease association (if any)	Presumed causative finding on ES	Carrier frequency (approximate, gnomAD)
18	chr22q11.–3 ‐ q12.1	chr22:25672585–25903543	0.231 (231 kb)	Het deletion	IGLL3P, LRP5L, CRYBB2P1, MIR6817	Bronchopulmonary dysplasia		1.03e‐2
19	chr22q11.–3 ‐ q12.1	chr22:25672585–25903543	0.231 (231 kb)	Het deletion	IGLL3P, LRP5L, CRYBB2P1, MIR6817	Bronchopulmonary dysplasia		1.03e‐2
20	chr22q11.23‐q12.1	chr22:25645676–25903543	0.238 (238 kb)	Het deletion	IGLL3P, LRP5L, CRYBB2P1, MIR6817	Bronchopulmonary dysplasia		1.03e‐2
21	chr22q11.–3 ‐ q12.1	chr22:25672585–25903543	0.231 (231 kb)	Het duplication	IGLL3P, LRP5L, CRYBB2P1, MIR6817	Bronchopulmonary dysplasia	Also has MYBPC3 (chr11p11.2) c.3791G>A p.C1264Y	1.30e‐2
22	chr9q12 ‐q21.11	chr9:65632517–71016040	5.38 Mb	Het duplication	Several (see Table S2, Supporting Information)			0
15	chr15q26.2	chr15:96869390–96950543	0.081 (81 kb)	Het deletion	NR2F2	(Involved in the hippocampus)	Also has a chrX duplication, indicative of XXY	4.60e‐5
23	chr15q26.2	chr15:96869390–96896882	0.027 (27 kb)	Het deletion	NR2F2	(Involved in the hippocampus)		4.60e‐5
24	chr11p13	chr11: 31172410–31279781	0.107 (107 kb)	Het deletion	DCDC1		Also has SCN4A:c.2045C>G, p.Ser682Trp, 2045/5511	9.70e‐5
25	chr13q12.12	chr13: 25165136–25327563	0.162 (162 kb)	Het duplication	TPTE2P6, ATP12A		MYBPC3 VUS‐FP c.3791G>A, p.Cys1264Tyr	1.38e‐4
26	chr14q11.2	chr14: 19794577–20421677	0.627 (627 kb)	Het duplication	LINC01296, DUXAP10, BMS1P22, BMS1P18, BMS1P17, POTEM, LOC100508046, OR11H2, OR4Q3, OR4M1, OR4N2, OR4K3, OR4K2, OR4K5, OR4K1		TCF4 VUS‐FP c.868T>A, p.Ser290Thr	0
27	chr15q15.3	chr15:43851548–43951301	0.997 (997 kb)	Het duplication	PPIP5K1, CKMT1B, STRC, CATSPER2, PPIP5K1P1‐CATSPER2		SCN1A LP c.2045G>T, p.Gly682Val	6.48e‐3

Two of the 53 cases with VUS, cases 15 and 23, had overlapping and uncharacterized VUS CNV duplications at 15q26.2 encompassing *NR2F2* (chr15: 96869390–96950543, 81 kb and chr15: 96869390–96896882, 27 kb). *NR2F2* is linked to hippocampal function,^[^ ^81^ ^]^ and with a pLi of 0.99, this gene is expressed in most tissues, including in the brain predominately in the hippocampus and amygdala. Although pathogenic variants in *NR2F2* are associated with congenital diaphragmatic hernia (CDH),^[^ ^82^ ^]^ multiple types of congenital heart defects,^[^ ^83^, ^84^ ^]^ and with 46,XX sex reversal,^[^ ^85^ ^]^ duplication of *NR2F2* is not known to be associated with a phenotype. Of note, case 15 (81 kb duplication) also had a complete duplication of the X chromosome noted above.

Four cases had CNVs at chr22q11.23–q12.1 (chr22:25672585–25903543) involving *IGLL3P, LRP5L, CRYBB2P1*, and *MIR6817* (three deletions and one duplication). This 22q11.23–q12.1 region is associated with bronchopulmonary dysplasia (BPD) in premature infants^[^ ^86^ ^]^ and in our cases is not likely to be related to SUDP, as our cases were not born prematurely. Two cases had duplications at 15q26.2 (chr15:96869390–96950543 and chr15:96869390–96896882) encompassing *NR2F2*. Loss of function and missense variants of *NR2F2* are associated with disease (46XX sex reversal, CDH,^[^ ^82^ ^]^ and cardiac defects^[^ ^84^ ^]^). However, there are no known disease associations with duplications of *NR2F2*.

#### 3.2.5 Genome‐Wide CNV Pathogenicity Analyses

The computed pathogenicity score was higher in the SUDP cohort compared to the control cohort for deletions (OR 1.24, 95%CI 1.11–1.31, *p* = 0.007) (**Table** **8**). When comparing the SUDP cohort deletion score to that of the ASD cohort, there was no significant difference. Removing bed sharing cases from the analysis did not alter the OR (OR 1.24, 95% CI 1.16–1.33 *p* = 0.03). The pathogenicity score was not significantly different between the SUDP cohort and controls or ASD cohort for duplications.

**Table 8:** Comparison of sum of 1/LOEUF pathogenicity score, SUDP vs. control, and ASD cohorts

	SUDP (*N* = 114) vs. controls		Removing bed sharing SUDP (*N* = 66) vs. controls		SUDP (*N* = 114) vs. ASD		Removing bed sharing or unknown if bed sharing SUDP (*N* = 66) vs. ASD	
Variable	OR [95%CI] *p*‐value		OR [95%CI] *p*‐value		OR [95%CI] *p*‐value		OR [95%CI] *p*‐value	
	DEL	DUP	DEL	DUP	DEL	DUP	DEL	DUP
Pathogenicity score	1.24 [1.11‐1.31] *p* = 0.007	n.s.	1.24 [1.16‐1.33] *p* = 0.03	n.s.	n.s.	n.s.	n.s.	n.s.

The pathogenicity score represents the sum of 1/LOEUF of all genes encompassed in CNVs identified in each individual. As sensitivity analysis, we removed cases found while bed‐sharing or if this information was not available. SUDP: Sudden Unexplained Death in Pediatrics; ASD: autism spectrum disorder; N: number; OR: odds ratio; 95%CI: 95% of confidence interval; DEL: deletions; DUP: duplications.

#### 3.2.6 Recurrent CNV Analyses

In our cohort, 3/114 SUDP cases (2.6%) harbored a deletion involving an interval associated with schizophrenia and/or other neurodevelopmental phenotypes (see Figure 1). Cases 6 and 7 both had chr15q11.2 deletions that are associated with schizophrenia, attention deficit hyperactivity disorder (ADHD), and ASD.^[^ ^65^, ^87^, ^88^, ^89^ ^]^ Case 1 had a *NRXN1* deletion, which likewise is associated with a wide spectrum of developmental and neuropsychiatric disorders, including intellectual disability, speech delay, ASD, hypotonia, and schizophrenia.^[^ ^90^ ^]^ We were interested in whether this rate of CNVs was higher than in ASD or control populations. However, we found no difference in the frequency of known neuropsychiatric CNVs in controls or ASD versus our SUDP cohort after FDR correction (**Tables** **9** and **10**).

**Table 9:** Comparison of known neuropsychiatric regions in SUDP and control cohorts

Neuropsychiatric associated regions	SUDP (*N* = 114) vs. controls OR [95%CI] *p*‐value	Removing bed sharing or unknown if bed‐sharing SUDP (*N* = 66) vs. controls OR [95%CI] *p*‐value
15q11.2 deletion	*N* = 2 vs. 69 4 [0.5‐16] *p* = 0.08 (p‐FDR = 0.22)	*N* = 2 vs. 69 7 [0.9‐29] *p* = 0.03 (p‐FDR = 0.09)
Deleted NRXN1	*N* = 1 vs. 16 9 [0.2‐60] *p* = 0.11 (p‐FDR = 0.22)	*N* = 1 vs. 16 16 [0.4‐105] *p* = 0.07 (p‐FDR = 0.12)
All deleted and duplicated regions	*N* = 3 vs. 302 1.4 [0.3‐4] *p* = 0.47 (p‐FDR = 0.47)	*N* = 3 vs. 302 2 [0.5‐8] *p* = 0.12 (p‐FDR = 0.12)
All deleted regions	*N* = 3 vs. 158 3 [0.5‐8] *p* = 0.10 (p‐FDR = 0.22)	*N* = 3 vs. 158 5 [1, 2, 3, 4, 5, 6, 7, 8, 9, 10, 11, 12, 13, 14, 15] *p* = 0.03 (p‐FDR = 0.09)
Deleted recurrent CNVs only	*N* = 2 vs. 130 2 [0.3‐8] *p* = 0.23 (p‐FDR = 0.28)	*N* = 2 vs. 130 4 [0.5‐15] *p* = 0.10 (p‐FDR = 0.12)
Deleted single genes only	*N* = 1 vs. 28 5 [0.1‐32] *p* = 0.18 (p‐FDR = 0.27)	*N* = 1 vs. 28 9 [0.2‐56] *p* = 0.11 (p‐FDR = 0.12)

Legend: None of these results remain significant after FDR correction. The recurrent CNVs category represents the neuropsychiatric regions occurring by non‐allelic homologous recombination identified in our cohorts. The single genes category represents the neuropsychiatric genes disrupted by a CNV identified in our cohorts. As sensitivity analysis, we removed cases found while bed‐sharing or if this information was not available. SUDP: Sudden Unexplained Death in Pediatrics; N: number; OR: odds ratio; 95%CI: 95% of confidence interval.

**Table 10:** Comparison of known neuropsychiatric regions in SUDP and ASD cohorts

Neuropsychiatric associated regions	SUDP (*N* = 114) vs. ASD OR [95%CI] *p*‐value	Removing bed sharing SUDP (*N* = 66) vs. ASD OR [95%CI] *p*‐value
15q11.2 deletion	*N* = 2 vs. 16 6 [0.7‐27] *p* = 0.05 (p‐FDR = 0.30)	*N* = 2 vs. 16 10 [1.2‐47] *p* = 0.02 (p‐FDR = 0.12)
Deleted NRXN1	*N* = 1 vs. 19 3 [0.1‐16] *p* = 0.33 (p‐FDR = 0.69)	*N* = 1 vs. 19 4 [0.1‐29] *p* = 0.21 (p‐FDR = 0.38)
All deleted and duplicated regions	*N* = 3 vs. 227 0.6 [0.1‐2] *p* = 0.63 (p‐FDR = 0.69)	*N* = 3 vs. 227 1 [0.2‐3] *p* = 0.75 (p‐FDR = 0.75)
All deleted regions	*N* = 3 vs. 113 1.3 [0.2‐4] *p* = 0.5 (p‐FDR = 0.69)	*N* = 3 vs. 113 2 [0.4‐7] *p* = 0.16 (p‐FDR = 0.38)
Deleted recurrent CNVs only	*N* = 2 vs. 83 1.2 [0.1‐4] *p* = 0.69 (p‐FDR = 0.69)	*N* = 2 vs. 83 2 [0.2‐8] *p* = 0.26 (p‐FDR = 0.38)
Deleted single genes only	*N* = 1 vs. 31 1.6 [0.04‐10] *p* = 0.48 (p‐FDR = 0.69)	*N* = 1 vs. 31 3 [0.1‐17] *p* = 0.32 (p‐FDR = 0.38)

Legend: None of these results remain significant after FDR correction. The recurrent CNVs category represents the neuropsychiatric regions occurring by non‐allelic homologous recombination identified in our cohorts. The single genes category represents the neuropsychiatric genes disrupted by a CNV identified in our cohorts. As sensitivity analysis, we removed cases found while bed‐sharing or if this information was not available. SUDP: Sudden Unexplained Death in Pediatrics; ASD: autism spectrum disorder; N: number; OR: odds ratio; 95%CI: 95% of confidence interval.

## 4 Discussion

While we and others have successfully identified potentially new genes and mechanisms for SUDP through the use of next‐generation sequencing,^[^ ^3^, ^4^, ^5^, ^7^, ^8^, ^56^, ^91^, ^92^ ^]^ assessment of structural variation in the genome in SUDP cases has not been extensively reported. Here we report a detailed CMA analysis of 116 well‐phenotyped SUDP cases, for which we additionally have completed ES analysis.^[^ ^5^ ^]^ We identified five cases (4.3%) with pathogenic CNVs and nine (7.8%) with CNVs categorized as VUS favoring pathogenic (total 12.1%). Additionally, we identified two cases of X chromosome duplication consistent with a 47,XXY karyotype and Klinefelter syndrome (2.9% of boys) and one with a 22.7 Mb region of LOH of the X chromosome suggesting possible UPD (0.8%).

Two cases with CNVs also had a reportable finding on ES (a pathogenic *SCN1A* variant in girl case 17 with LOH on chromosome X, and a VUS favoring pathogenic *PDHA1* variant in boy case 8 with a duplication on chr13q34).^[^ ^5^ ^]^ While these ES variants call into question the relevance of the CMA findings in these cases, we cannot rule out the possibility of an additive effect between the CMA and ES findings, particularly since the mechanisms contributing directly to death are not known. Similarly, of the 53 cases (46%) with one or more CNVs categorized as VUS, five had a finding on ES analysis that potentially explained the cause of death.

In the only other published study of CNVs in a cohort of SIDS cases, CNVs were detected in 3 of 27 cases (11%),^[^ ^38^ ^]^ close to our rate of 12.1%. We did not detect any deletions/duplications in the regions described in that study (a 6p22 deletion, an 8q24.3‐qter duplication, and a 22q13.3‐qter deletion).^[^ ^38^ ^]^

### 4.1 Pathogenic Findings and Structural Variants

The varied nature of the phenotypes typically associated with the CNVs detected in our cohort, and the absence or inability to verify many of these features in our cases, raises the question of whether the SUDP deaths in our cohort were related to the CNVs identified. We were unable to perform CMAs on the parents to determine if these CNVs were de novo or inherited, which presents a limitation in assessing pathogenicity.

Several of the CNVs that we detected are associated with neurodevelopmental phenotypes including seizures, ASD, developmental delay, ADHD, and schizophrenia. These include Klinefelter syndrome,^[^ ^93^, ^94^, ^95^ ^]^ *NRXN1* deletion,^[^ ^96^, ^97^, ^98^ ^]^ and 15q11.2 deletion syndrome.^[^ ^99^ ^]^ The association with seizures is of particular interest as there is evidence from our previous studies, as well as others, that seizures and epilepsy are linked to SIDS and SUDC, perhaps as a direct cause of death or perhaps because of a shared pathogenesis due to a heightened susceptibility to other conditions such as arrhythmogenic sudden cardiac death or other mechanism.^[^ ^7^, ^41^, ^96^, ^100^, ^101^, ^102^, ^103^, ^104^ ^]^

In Klinefelter syndrome, seizures (febrile, generalized tonic‐clonic, focal impaired awareness seizures, and absence) have an estimated prevalence of 5–17%.^[^ ^76^, ^93^ ^]^ The two boys with 47,XXY (6 weeks and 3 months of age) were not known to have Klinefelter syndrome prior to birth and had no history of seizures. Additionally, Klinefelter syndrome is associated with an increased risk of adverse fetal and neonatal outcomes, and the rate of neonatal death is 9.5 times higher in Klinefelter syndrome compared to the general population (1.9% vs. 0.2% *p* < 0.0001), and rates of infant death are more than 50 times higher (5.8% vs. 0.1%, *p* < 0.0001).^[^ ^105^ ^]^ The mechanisms for the increased rate of adverse events and deaths in Klinefelter syndrome are not yet determined.

Deletions of *NRXN1* and the 15q11.2 deletion syndrome are also associated with seizures.^[^ ^106^ ^]^ To be clear, we were not aware of a history of epilepsy or seizures antecedent to death in our SUDP cases with these findings, but together they raise the question of whether seizure‐related mechanisms may have played a role in death, as has been supported by prior evidence from other cases.^[^ ^107^ ^]^

Klinefelter syndrome, *NRXN1* deletion, and 15q11.2 deletion syndrome are associated with ASD and ADHD;^[^ ^93^, ^94^, ^95^ ^]^ *NRXN1* deletion and 15q11.2 deletion syndrome^[^ ^65^, ^99^, ^108^ ^]^ are also associated with developmental delay and schizophrenia. Both *NRXN1* deletion and 15q11.2 deletion syndrome have incomplete penetrance and variable expressivity. In one study of 15q11.2 deletion syndrome, 81.2% of cases inherited the CNV from one of the parents.^[^ ^88^ ^]^ Studies have shown that adults with a 15q11.2 deletion have a ≈90% likelihood of a normal phenotype.^[^ ^67^, ^109^ ^]^ As the infants and children in this study with 15q11.2 deletions died prior to the age when psychiatric symptoms would have presented, this makes the interpretation of the impact of the 15q11.2 deletion challenging.

### 4.2 VUS Findings

The relationship to SUDP in the cases with a VUS is unclear. While none of the cases with a CNV interpreted as “pathogenic” had a presumed causative finding on ES, one case in 9 with CNVs interpreted as “VUS favoring pathogenic” had a presumed causative finding on ES, and five of 53 cases with CNVs interpreted as “VUS” had a presumed causative finding on ES (9.4%).

### 4.3 CNVs over 1 Mb

While CNVs larger than 500 kb are found in around 5–10% of individuals,^[^ ^110^ ^]^ they are found in almost 25% of patients with intellectual disability.^[^ ^111^, ^112^ ^]^ Variants greater than 1 Mb are only found in 1–2% of the population.^[^ ^110^ ^]^ In this cohort, 18% of the total cohort had a CNV larger than 1 Mb. Although the correlation drawn between CNV size and its clinical significance is often true, very large CNVs can be benign in nature^[^ ^110^ ^]^ and the genome is more polymorphic than previously thought. Therefore, this finding is difficult to interpret.

### 4.4 Increased Pathogenicity in CNV Deletions

Interestingly, we observed a significantly increased pathogenicity score, indicated by the sum of 1/LOEUF, for deletions in our SUDP cohort compared to control cohorts, but not compared to an ASD cohort. These results suggest an overlap between the genetic burden observed in both SUDP and ASD populations. Such similarities are in line with findings from a recent study reporting that genes altered in SUDC are also enriched in lists of genes previously associated with neurodevelopmental disorder, and more particularly with ASD.^[^ ^8^ ^]^ However, since our cohort was of modest size, replication of this finding with larger and independent samples is paramount. Reproducing such analyses by using a non‐neurodevelopmental disorder (e.g., populations with asthma or cancer) as a comparison group would provide more detail on the nature of the CNV burden.

### 4.5 Limitations

One limitation of our study is the relatively small sample size of our cohort, which requires that we interpret the findings with caution. Second, due to the nature of how our cohort was ascertained and consented, we were not able to conduct CMA analyses on the parents of the cases or interview parents directly to obtain additional phenotypic details or family history. Thus, we are unable to determine if CNVs detected were de novo or inherited, which would impact our assessment of their pathogenicity and impact. Another limitation is the completeness of available phenotypic data that is restricted to autopsy material and available medical records. Particular phenotypes, such as cortical thinning in 15q11.2 deletions, are not routinely assessed in autopsy and are not standard phenotyping for well‐baby medical visits. Thus, we cannot determine whether the cases had some of the phenotypic characteristics associated with the observed CNV intervals. Additionally, this CMA study focuses on children aged 28 months and younger (average age at death, 5.7 months) and findings may not be generalizable for older children. Finally, the Agilent 4 × 180 array used in this study is unable to detect complex rearrangements, repeat expansions, and balanced translocations.^[^ ^113^ ^]^ Thus structural variation in these cases would have been missed.

### 4.6 Conclusions and Recommendations

Although we did not find any CNVs that definitively explain the cause of death in our SUDP cohort, our findings suggest new avenues to investigate intrinsic vulnerabilities in the SUDP population. Notably, the pathogenicity score was significantly increased for deletions in comparison to a control database and was similar to the ASD cohort. In addition, several CNVs are associated with neurodevelopmental phenotypes, including two cases with 47,XXY. These findings raise the question of whether the neurodevelopmental or some other consequence of abnormal gene dosage from these CNVs has contributed to death in these cases. In addition, our findings lead to the intriguing question of whether the same causative factors that result in disorders including intellectual disability, ASD, ADHD, and epilepsy (as we have previously reported in the context of SNVs),^[^ ^88^, ^106^, ^114^ ^]^ may also be involved in terminal mechanism contributing to SUDP. The role of neuropsychiatric disease‐associated CNVs in SUDP warrants further investigation.

Since ES does yet not adequately detect CNVs, when sequencing results are inconclusive, an assessment for CNVs by CMA may provide some answers for families and valuable information on CNVs observed in SUDP cases. The emerging role of CNVs in SUDP warrants further study. In particular, we advocate for dedicated CNV analysis with CMA in cases for which ES is unrevealing, including parental CNV assessment so that de novo versus inherited status can be determined. Future mechanistic studies will allow us to understand the role that specific CNVs may play in sudden death. In concert with past reports of ES findings in SUDP from our group and others, the findings presented here support comprehensive assessment with ES and CMA analysis for all cases of SUDP.

## Conflict of Interest

The authors declare no conflict of interest.

## Author Contributions

C.A.B., E.D., R.D.G., and I.A.H. contributed to the conceptualization and primary writing. C.A.B., E.D., R.L.H., H.Y.K., A.H., C.K., S.A., E.A.H., S.O.V., M.H.W., S.J., A.H.P., R.D.G., and I.A.H. contributed to data collection, analysis and interpretation of results, discussion, review, and editing of the manuscript.

## Supporting information

Supporting Information

## Data Availability

The data that support the findings of this study are available on request from the corresponding author. The data are not publicly available due to privacy or ethical restrictions.
