# Supplementary material for: Copy Number Variation and Structural Genomic Findings in 116 Cases of Sudden Unexplained Death between 1 and 28 Months of Age
Source: Adv Genet (Hoboken). 2022 Nov 7;4(1):2200012. doi: 10.1002/ggn2.202200012 (PMC10000288; doi:10.1002/ggn2.202200012)
Supplement: Supplementary file 1 — Supporting Information [file GGN2-4-2200012-s001.pdf]

Supplemental Table 1: Recurrent neurodevelopmental or neuropsychiatric-associated loci and genes investigated (GRCh37/hg19)

Locus	CHR	START	STOP
**1p36**	chr1	1	2500000
**1q21.1 TAR**	chr1	145394955	145807817
**1q21.1 distal+TAR**	chr1	145394955	147394444
**1q21.1 distal**	chr1	146527987	147394444
NRXN1	chr2	50145643	51259674
**2q11.2**	chr2	96742409	97677516
**2q13**	chr2	111394040	112012649
**2q21.1**	chr2	131481308	131930677
**2q37**	chr2	239716679	243199373
**3q29**	chr3	195720167	197354826
**4p16.3**	chr4	1552030	2091303
**5q35**	chr5	175720924	177052594
SIM1	chr6	100836750	100911811
**7q11.23 WBS**	chr7	72744915	74142892
**7q11.23 distal**	chr7	75138294	76064412
**8p23.1**	chr8	8098990	11872558
**9q34**	chr9	140513444	140730578
**10q11.21q11.23**	chr10	49390199	51058796
**10q22q23**	chr10	82045472	88931651
**11p11.2**	chr11	43940000	46020000
CRYL1	chr13	20977806	21100012

**13q12.12**	chr13	23555358	24884622
**15q11.2**	chr15	22805313	23094530
**15q11.2q12**	chr15	22805313	28390339
**15q13.1q13.2 BP3-BP4**	chr15	29161368	30375967
**15q13.1q13.3 BP3-BP5**	chr15	29161368	32462776
**15q13.3 BP4-BP5**	chr15	31080645	32462776
**15q24**	chr15	72900171	78151253
**15q25.2**	chr15	83219735	85722039
CREBBP	chr13	3775056	3930121
**16p13.11**	chr16	15511655	16293689
**16p12.1**	chr16	21950135	22431889
**16p11.2 distal**	chr16	28823196	29046783
**16p11.2 distal+proximal**	chr16	28823196	30200773
**16p11.2 proximal**	chr16	29650840	30200773
**16p11.2p12.1**	chr16	21596415	28347808
YWHAE	chr17	1247834	1303556
PAFAH1B1	chr17	2496923	2588909
**17p12**	chr17	14141387	15426961
**17p11.2**	chr17	16812771	20211017
**17q11.2 *NF1***	chr17	29107491	30265075
**17q12**	chr17	34815904	36217432
**17q21.31**	chr17	43705356	44164691
**17q23.1q23.2**	chr17	58302389	60289141

**22q11.2 distal**	chr22	21920127	23653646
**22q11.2 proximal**	chr22	19037332	21466726
SHANK3	chr22	51113070	51171640

Legend: List of recurrent loci and genes from Kendall *et al.*, *British Journal of Psychiatry* (2019).[1] Coordinates are presented in hg19 (Homo sapiens (human) genome assembly GRCh37) from Genome Reference Consortium; CHR: chromosome; BP: break points; WBS: William-Beuren syndrome

Supplemental Table 2: CNVs over 1Mb

ID	Chr	Cytoband	Start	Stop	Type	Genes	Size
28	chr16	p11.2	32279544	33773134	Loss	LOC390705, TP53TG3E, TP53TG3, TP53TG3B, TP53TG3F, TP53TG3C, SLC6A10P, ENPP7P13	1493590
29	chr16	p11.2	32279544	33489524	Loss	LOC390705, TP53TG3E, TP53TG3, TP53TG3B, TP53TG3F, TP53TG3C, SLC6A10P	1209980
30	chr16	p11.2	32279544	33489524	Loss	LOC390705, TP53TG3E, TP53TG3, TP53TG3B, TP53TG3F, TP53TG3C, SLC6A10P	1209980
15	chrX	p22.33	60701	2646815	Gain	PLCXD1, GTPBP6, LINC00685, PPP2R3B, SHOX, CRLF2, CSF2RA, MIR3690, IL3RA, SLC25A6, LINC00106, ASMTL-AS1, ASMTL, P2RY8, AKAP17A, ASMT, DHRSX, ZBED1, MIR6089, CD99P1, LINC00102, CD99	2586114

						XG, XGY2, GYG2, ARSD, ARSD-AS1, ARSE, ARSH, ARSF, LINC01546, MXRA5, SNORA48B, PRKX, PRKX-AS1, LOC389906, FAM239B, FAM239A, LOC101928201, NLGN4X, LOC105373156, MIR4770, VCX3A, PUDP, STS, MIR4767, VCX, PNPLA4, MIR651, VCX2, VCX3B, ANOS1, FAM9A, FAM9B, TBL1X, GPR143, SHROOM2, CLDN34, WWC3, CLCN4, MID1, HCCS, ARHGAP6, AMELX, MIR548AX, MSL3, FRMPD4, PRPS2, TLR7, TLR8-AS1, TLR8, TMSB4X, FAM9C, LINC02154, GS1-600G8.3, ATXN3L, LINC01203, EGFL6, MIR6086, TCEANC, RAB9A, TRAPPC2, OFD1, GPM6B, GEMIN8, UBE2E4P, GLRA2, FANCB, MOSPD2, ASB9, ASB11, PIGA, VEGFD, PIR-FIGF, PIR, BMX, ACE2, GS1-594A7.3, CLTRN, CA5BP1, CA5B, INE2, ZRSR2, AP1S2, GRPR, MAGEB17, CTPS2, MIR548AM, S100G, SYAP1, TXLNG, RBBP7, REPS2, NHS, MIR4768, LOC101928389, NHS-AS1, SCML1, RAI2, LINC01456, BEND2, SCML2, CDKL5, RS1, PPEF1-AS1, PPEF1, PHKA2-AS1, PHKA2, ADGRG2, PDHA1, MAP3K15, SH3KBP1, BCLAF3, LOC729609, MAP7D2, MIR23C, EIF1AX, SCARNA9L, EIF1AX-AS1, RPS6KA3, CNKSR2, KLHL34, SMPX, MBTPS2, YY2, SMS, PHEX, PHEX-AS1, PTCHD1-AS, ZNF645, DDX53, PTCHD1, PRDX4, ACOT9, SAT1, APOO, CXorf58, KLHL15, EIF2S3, ZFX-AS1, ZFX, SUPT20HL2, SUPT20HL1, PDK3, PCYT1B, PCYT1B-AS1, POLA1, SCARNA23, ARX, MAGEB18, MAGEB6, MAGEB5, VENTXP1, PPP4R3C, DCAF8L2, MAGEB10, DCAF8L1, MIR6134, IL1RAPL1, MIR4666B, MAGEB2, MAGEB3, MAGEB4, MAGEB1, NR0B1, CXorf21, GK, TAB3, TAB3-AS1, FTHL17, DMD, MIR3915, MIR548F5, FAM47A, TMEM47, FAM47B, MAGEB16, CFAP47, LOC101928627, FAM47C, FTH1P18, PRRG1, LANCL3, XK, CYBB, DYNLT3, HYPM, SYTL5, MIR548AJ2, SRPX, RPGR, OTC, TSPAN7, MID1IP1-AS1, MID1IP1, LINC01281, LINC01282, MIR3937, BCOR, LOC101927476, ATP6AP2, MPC1L, CXorf38, MED14, MED14OS, LOC100132831, USP9X, LOC105373185, MIR7641-2, DDX3X, NYX, CASK, GPR34, GPR82, PPP1R2P9, PINCR, MAOA, MAOB, NDP, NDP-AS1, EFHC2, FUNDC1, DUSP21, KDM6A, CXorf36, LINC01204, LOC392452, MIR221, MIR222, LOC401585, LINC01186, KRBOX4, ZNF674, ZNF674-AS1, CHST7, SLC9A7, RP2, LINC01545, JADE3, RGN, NDUFB11, RBM10, UBA1, INE1, CDK16, USP11, ZNF157, SNORA11C, ZNF41, LINC01560, ARAF, SYN1, TIMP1, MIR4769, CFP, ELK1, UXT, UXT-AS1, CXXC1P1, ZNF81, ZNF182, SPACA5, SPACA5B, ZNF630-AS1, ZNF630, SSX6, SSX5, SSX1, SSX9, SSX3, SSX4B, SSX4, SLC38A5, FTSJ1, LOC101927635, [illegible]	

						SPIN4, LINC01278, ARHGEF9, ARHGEF9-IT1, MIR1468, AMER1, ASB12, MTMR8, ZC4H2, ZC3H12B, LAS1L, FRMD8P1, MSN, MIR223, VSIG4, HEPH, EDA2R, AR, OPHN1, YIPF6, STARD8, EFNB1, PJA1, LINC00269, FAM155B, EDA, MIR676, AWAT2, OTUD6A, IGBP1, DGAT2L6, AWAT1, P2RY4, ARR3, RAB41, PDZD11, KIF4A, GDPD2, DLG3, DLG3-AS1, TEX11, SNORD3E, SLC7A3, SNX12, FOXO4, CXorf65, IL2RG, MED12, NLGN3, BCYRN1, GJB1, ZMYM3, NONO, ITGB1BP2, TAF1, INGX, OGT, GCNA, CXCR3, LOC100129291, LOC101059915, LOC100132741, LINC00891, CXorf49B, CXorf49, NHSL2, RPS26P11, RTL5, FLJ44635, PIN4, ERCC6L, RPS4X, CITED1, HDAC8, PHKA1, PHKA1-AS1, FAM236B, FAM236A, FAM236D, FAM236C, DMRTC1, DMRTC1B, FAM226B, FAM226A, PABPC1L2B-AS1, PABPC1L2B, PABPC1L2A, NAP1L6, NAP1L2, CDX4, MAP2K4P1, CHIC1, TSIX, XIST, JPX, FTX, MIR421, MIR374B, MIR374C, MIR545, MIR374A, ZCCHC13, SLC16A2, RLIM, NEXMIF, ABCB7, UPRT, ZDHHC15, TTC3P1, MAGEE2, PBDC1, MAGEE1, MIR325HG, MIR384, MIR325, FGF16, ATRX, MAGT1, COX7B, ATP7A, PGAM4, PGK1, TAF9B, CYSLTR1, RTL3, LPAR4, MIR4328, P2RY10, GPR174, ITM2A, TBX22, CHMP1B2P, FAM46D, BRWD3, HMGN5, SH3BGRL, POU3F4, CYLC1, RPS6KA6, MIR548I4, HDX, UBE2DNL, APOOL, SATL1, LOC101928128, ZNF711, POF1B, MIR1321, CHM, MIR361, DACH2, KLHL4, CPXCR1, TGIF2LX, PABPC5-AS1, PABPC5, PCDH11X, MIR4454, NAP1L3, FAM133A, MIR548M, BRDTP1, DIAPH2, RPA4, DIAPH2-AS1, XRCC6P5, PCDH19, TNMD, TSPAN6, SRPX2, SYTL4, CSTF2, NOX1, XKRX, ARL13A, TRMT2B, TMEM35A, CENPI, DRP2, TAF7L, TIMM8A, BTK, RPL36A, RPL36A-HNRNPH2, GLA, HNRNPH2, ARMCX4, ARMCX1, ARMCX6, ARMCX3, ARMCX2, NXF5, ZMAT1, TCEAL2, TCEAL6, BEX5, TCP11X2, NXF2B, NXF2, TMSB15A, NXF4, ARMCX5, ARMCX5-GPRASP2, GPRASP1, GPRASP2, BHLHB9, LINC00630, RAB40AL, BEX1, NXF3, BEX4, TCEAL8, TCEAL5, BEX2, TCEAL7, TCEAL9, BEX3, RAB40A, LOC105373300, TCEAL4, TCEAL3, TCEAL1, MORF4L2, MORF4L2-AS1, GLRA4, TMEM31, RAB9B, PLP1, LOC100128594, TMSB15B, H2BFXP, LOC100101478, H2BFWT, H2BFM, SLC25A53, ZCCHC18, LOC286437, FAM199X, ESX1, IL1RAPL2, TEX13A, NRK, SERPINA7, MUM1L1, CXorf57, MIR548AN, RNF128, TBC1D8B, RIPPLY1, CLDN2, MORC4, RBM41, NUP62CL, PIH1D3, FRMPD3-AS1, FRMPD3, PRPS1, TSC22D3, NCBP2L, MID2, LOC101928335, TEX13B, VSIG1, PSMD10, ATG4A, COL4A6, COL4A5, IRS4, [illegible]	

	chrY	p11.32 - p11.2	10701	6094549	Gain	PLCXD1, GTPBP6, LINC00685, PPP2R3B, SHOX, CRLF2, CSF2RA, MIR3690, IL3RA, SLC25A6, LINC00106, ASMTL-AS1, ASMTL, P2RY8, AKAP17A, ASMT, DHRSX, ZBED1, MIR6089, CD99P1, CD99, XGY2, SRY, RPS4Y1, ZFY, ZFYAS1, LINC00278, TGIF2LY, PCDH11Y	6083848
31	chr16	p11.2	32279544	34260115	Gain	LOC390705, TP53TG3E, TP53TG3, TP53TG3B, TP53TG3F, TP53TG3C, SLC6A10P, ENPP7P13	1980571
32	chr16	p11.2	32279544	33489524	Loss	LOC390705, TP53TG3E, TP53TG3, TP53TG3B, TP53TG3F, TP53TG3C, SLC6A10P	1209980
33	chr9	p13.1 - p11.2	38869901	44089803	Gain	CNTNAP3, SPATA31A1, FAM74A1, ZNF658B, LOC105379450, SPATA31A3, FAM74A3, ZNF658, SPATA31A5, SPATA31A7, FAM74A6, GLIDR, FGF7P3, LOC102724238, LOC554249, ANKRD20A2, ANKRD20A3, FAM95B1, LOC105379252, GXYLT1P3, FOXD4L4, LOC101928381, LOC101928195, LOC101929583, LOC286297, AQP7P3, LOC642929, FAM74A7, SPATA31A6, CNTNAP3B, CNTNAP3P2	5219902
34	chr16	p11.2	32279544	33773134	Loss	LOC390705, TP53TG3E, TP53TG3, TP53TG3B, TP53TG3F, TP53TG3C, SLC6A10P, ENPP7P13	1493590
22	chr9	q12 - q21.11	65632517	71016040	Gain	LINC01410, LOC100132249, PTGER4P2-CDK2AP2P2, LOC403323, LOC728673, LOC101928381, LOC101928195, LOC101929583, LOC286297, AQP7P1, LOC102724580, BMS1P14, LOC102723709, FAM27E3, LOC105379807, FAM27B, ANKRD20A1, ANKRD20A3, LINC00537, MIR4477A, MIR4477B, FRG1JP, LOC102725126, FRG1HP, MIR1299, PGM5P2, LOC440896, FOXD4L6, CBWD6, CBWD5, ANKRD20A4-ANKRD20A20P, ANKRD20A4, LOC105379252, LOC100133920, FOXD4L5, FOXD4L4, CBWD3, FOXD4L3, PGM5-AS1, PGM5	5383523
	chr16	p11.2	32279544	33563699	Loss	LOC390705, TP53TG3E, TP53TG3, TP53TG3B, TP53TG3F, TP53TG3C, SLC6A10P	1284155
35	chr16	p11.2	32279544	33563699	Loss	LOC390705, TP53TG3E, TP53TG3, TP53TG3B, TP53TG3F, TP53TG3C, SLC6A10P	1284155

36	chr14	q11.2	19376762	20439064	Loss	OR11H12, LINC02297, POTEG, LOC101929572, POTEH-AS1, DUXAP10, LINC01296, BMS1P18, BMS1P17, BMS1P22, POTEM, LOC100508046, OR11H2, OR4Q3, OR4M1, OR4N2, OR4K3, OR4K2, OR4K5, OR4K1	1062302
	chr16	p11.2	32279544	33563699	Loss	LOC390705, TP53TG3E, TP53TG3, TP53TG3B, TP53TG3F, TP53TG3C, SLC6A10P	1284155

[illegible]

16							XG, GYG2, ARSD, ARSD-AS1, ARSE, ARSH, ARSF, LINC01546, MXRA5, SNORA48B, PRKX, PRKX-AS1, LOC389906, FAM239B, FAM239A, LOC101928201, NLGN4X, LOC105373156, MIR4770, VCX3A, PUDP, STS, MIR4767, VCX, PNPLA4, MIR651, VCX2, VCX3B, ANOS1, FAM9A, FAM9B, TBL1X, GPR143, SHROOM2, CLDN34, WWC3, CLCN4, MID1, HCCS, ARHGAP6, AMELX, MIR548AX, MSL3, FRMPD4, PRPS2, TLR7, TLR8-AS1, TLR8, TMSB4X, FAM9C, LINC02154, GS1-600G8.3, ATXN3L, LINC01203, EGFL6, MIR6086, TCEANC, RAB9A, TRAPPC2, OFD1, GPM6B, GEMIN8, UBE2E4P, GLRA2, FANCB, MOSPD2, ASB9, ASB11, PIGA, VEGFD, PIR-FIGF, PIR, BMX, ACE2, GS1-594A7.3, CLTRN, CA5BP1, CA5B, INE2, ZRSR2, AP1S2, GRPR, MAGEB17, CTPS2, MIR548AM, S100G, SYAP1, TXLNG, RBBP7, REPS2, NHS, MIR4768, LOC101928389, NHS-AS1, SCML1, RAI2, LINC01456, BEND2, SCML2, CDKL5, RS1, PPEF1-AS1, PPEF1, PHKA2-AS1, PHKA2, ADGRG2, PDHA1, MAP3K15, SH3KBP1, BCLAF3, LOC729609, MAP7D2, MIR23C, EIF1AX, SCARNA9L, EIF1AX-AS1, RPS6KA3, CNKSR2, KLHL34, SMPX, MBTPS2, YY2, SMS, PHEX, PHEX-AS1, PTCHD1-AS, ZNF645, DDX53,<br>PTCHD1, PRDX4, ACOT9, SAT1, APOO, CXorf58, KLHL15, EIF2S3, ZFX-AS1, ZFX, SUPT20HL2, SUPT20HL1, PDK3, PCYT1B, PCYT1B-AS1, POLA1, SCARNA23, ARX, MAGEB18, MAGEB6, MAGEB5, VENTXP1, PPP4R3C, DCAF8L2, MAGEB10, DCAF8L1, MIR6134, IL1RAPL1, MIR4666B, MAGEB2, MAGEB3, MAGEB4, MAGEB1, NR0B1, CXorf21, GK, TAB3, TAB3-AS1, FTHL17, DMD, MIR3915, MIR548F5, FAM47A, TMEM47, FAM47B, MAGEB16, CFAP47, LOC101928627,<br>FAM47C, FTH1P18, PRRG1, LANCL3, XK, CYBB, DYNLT3, HYPM, SYTL5, MIR548AJ2, SRPX, RPGR, OTC, TSPAN7, MID1IP1-AS1, MID1IP1, LINC01281, LINC01282, MIR3937, BCOR, LOC101927476, ATP6AP2, MPC1L, CXorf38, MED14, MED14OS, LOC100132831, USP9X, LOC105373185, MIR7641-2, DDX3X, NYX, CASK, GPR34, GPR82, PPP1R2P9, PINCR, MAOA, MAOB, NDP, NDP-AS1, EFHC2, FUNDC1, DUSP21, KDM6A, CXorf36, LINC01204, LOC392452, MIR221, MIR222, LOC401585, LINC01186, KRBOX4, ZNF674,	

						ZNF674-AS1, CHST7, SLC9A7, RP2, LINC01545, JADE3, RGN, NDUFB11, RBM10, UBA1, INE1, CDK16, USP11, ZNF157, SNORA11C, ZNF41, LINC01560, ARAF, SYN1, TIMP1, MIR4769, CFP, ELK1, UXT, UXT-AS1, CXXC1P1, ZNF81, ZNF182, SPACA5, SPACA5B, ZNF630-AS1, ZNF630, SSX6, SSX5, SSX1, SSX9, SSX3, SSX4B, SSX4, SLC38A5, FTSJ1, LOC101927635,	

						SPIN4, LINC01278, ARHGEF9, ARHGEF9-IT1, MIR1468, AMER1, ASB12, MTMR8, ZC4H2, ZC3H12B, LAS1L, FRMD8P1, MSN, MIR223, VSIG4, HEPH, EDA2R, AR, OPHN1, YIPF6, STARD8, EFNB1, PJA1, LINC00269, FAM155B, EDA, MIR676, AWAT2, OTUD6A, IGBP1, DGAT2L6, AWAT1, P2RY4, ARR3, RAB41, PDZD11, KIF4A, GDPD2, DLG3, DLG3-AS1, TEX11, SNORD3E, SLC7A3, SNX12, FOXO4, CXorf65, IL2RG, MED12, NLGN3, BCYRN1, GJB1, ZMYM3, NONO, ITGB1BP2, TAF1, INGX, OGT, GCNA, CXCR3, LOC100129291, LOC101059915, LOC100132741, LINC00891, CXorf49B, CXorf49, NHSL2, RPS26P11, RTL5, FLJ44635, PIN4, ERCC6L, RPS4X, CITED1, HDAC8, PHKA1, PHKA1-AS1, FAM236B, FAM236A, FAM236D, FAM236C, DMRTC1, DMRTC1B, FAM226B, FAM226A, PABPC1L2B-AS1, PABPC1L2B, PABPC1L2A, NAP1L6, NAP1L2, CDX4, MAP2K4P1, CHIC1, TSIX, XIST, JPX, FTX, MIR421, MIR374B, MIR374C, MIR545, MIR374A, ZCCHC13, SLC16A2, RLIM, NEXMIF, ABCB7, UPRT, ZDHHC15, TTC3P1, MAGEE2, PBDC1, MAGEE1, MIR325HG, MIR384, MIR325, FGF16, ATRX, MAGT1, COX7B, ATP7A, PGAM4, PGK1, TAF9B, CYSLTR1, RTL3, LPAR4, MIR4328, P2RY10, GPR174, ITM2A, TBX22, CHMP1B2P, FAM46D, BRWD3, HMGN5, SH3BGRL, POU3F4, CYLC1, RPS6KA6, MIR548I4, HDX, UBE2DNL, APOOL, SATL1, LOC101928128, ZNF711, POF1B, MIR1321, CHM, MIR361, DACH2, KLHL4, CPXCR1, TGIF2LX, PABPC5-AS1, PABPC5, PCDH11X, MIR4454, NAP1L3, FAM133A, MIR548M, BRDTP1, DIAPH2, RPA4, DIAPH2-AS1, XRCC6P5, PCDH19, TNMD, TSPAN6, SRPX2, SYTL4, CSTF2, NOX1, XKRX, ARL13A, TRMT2B, TMEM35A, CENPI, DRP2, TAF7L, TIMM8A, BTK, RPL36A, RPL36A-HNRNPH2, GLA, HNRNPH2, ARMCX4, ARMCX1, ARMCX6, ARMCX3, ARMCX2, NXF5, ZMAT1, TCEAL2, TCEAL6, BEX5, TCP11X2, NXF2B, NXF2, TMSB15A, NXF4, ARMCX5, ARMCX5-GPRASP2, GPRASP1, GPRASP2, BHLHB9, LINC00630, RAB40AL, BEX1, NXF3, BEX4, TCEAL8, TCEAL5, BEX2, TCEAL7, TCEAL9, BEX3, RAB40A, LOC105373300, TCEAL4, TCEAL3, TCEAL1, MORF4L2, MORF4L2-AS1, GLRA4, TMEM31, RAB9B, PLP1, LOC100128594, TMSB15B, H2BFXP, LOC100101478, H2BFWT, H2BFM, SLC25A53, ZCCHC18, LOC286437, FAM199X, ESX1, IL1RAPL2, TEX13A, NRK, SERPINA7, MUM1L1, CXorf57, MIR548AN, RNF128, TBC1D8B, RIPPLY1, CLDN2, MORC4, RBM41, NUP62CL, PIH1D3, FRMPD3-AS1, FRMPD3, PRPS1, TSC22D3, NCBP2L, MID2, LOC101928335, TEX13B, VSIG1, PSMD10, ATG4A, COL4A6, COL4A5, IRS4, [illegible]	

37	chr14	q11.2	19376762	20421677	Gain	OR11H12, LINC02297, POTEG, LOC101929572, POTEH-AS1, DUXAP10, LINC01296, BMS1P18, BMS1P17, BMS1P22, POTEM, LOC100508046, OR11H2, OR4Q3, OR4M1, OR4N2, OR4K3, OR4K2, OR4K5, OR4K1	1044915
38	chr5	q13.2	68849594	70636824	Loss	OCLN, GTF2H2C_2, GTF2H2C, GUSBP3, LOC653080, SERF1A, SERF1B, SMN2, SMN1, SMA4, GTF2H2B, SMA5, LOC441081, GUSBP9, NAIP, GTF2H2, LOC647859, SNORD13B-2, SNORD13B-1, LINC02197	1787230
	chrY	p11.2 - q11.21	9769512	13848200	Gain	none	4078688
39	chr16	p11.2 - p11.1	33604409	34777244	Gain	UBE2MP1, LINC01566, FRG2DP, TP53TG3HP	1172835
40	chr16	p11.2	32279544	33563699	Loss	LOC390705, TP53TG3E, TP53TG3, TP53TG3B, TP53TG3F, TP53TG3C, SLC6A10P	1284155
41	chrY	p11.32 - p11.2	2006987	6063918	Gain	DHRSX, ZBED1, MIR6089, CD99P1, CD99, XGY2, SRY, RPS4Y1, ZFY, ZFY-AS1, LINC00278, TGIF2LY, PCDH11Y	4056931
42	chr16	p11.2 - p11.1	32335047	34743643	Gain	TP53TG3E, TP53TG3, TP53TG3B, TP53TG3F, TP53TG3C, SLC6A10P, LOC390705, ENPP7P13, UBE2MP1, LINC01566, FRG2DP, TP53TG3HP	2408596
43	chr14	q11.2	19376762	20421677	Loss	OR11H12, LINC02297, POTEG, LOC101929572, POTEH-AS1, DUXAP10, LINC01296, BMS1P18, BMS1P17, BMS1P22, POTEM, LOC100508046, OR11H2, OR4Q3, OR4M1, OR4N2, OR4K3, OR4K2, OR4K5, OR4K1	1044915
44	chr16	p11.2 - p11.1	33604409	34827826	Gain	UBE2MP1, LINC01566, FRG2DP, TP53TG3HP	1223417
45	chr16	p11.2 - p11.1	32279544	34860767	Gain	LOC390705, TP53TG3E, TP53TG3, TP53TG3B, TP53TG3F, TP53TG3C, SLC6A10P, ENPP7P13, UBE2MP1, LINC01566, FRG2DP, TP53TG3HP	2581223
